# Comparative analysis of the seed microbiome in four major oilseed crops (rapeseed, sunflower, soybean, sesame) reveals host-specific assembly and potential application of seed core microbes

**DOI:** 10.3389/fpls.2026.1721916

**Published:** 2026-01-26

**Authors:** Yao Yao, Lelin Zhao, Yun Zhang, Ailing Duan, Yuanxue Yang, Aiyu Wang, Chao Xue, Jianhua Zhang, Ming Zhao

**Affiliations:** 1 Institute of Industrial Crops, Shandong Academy of Agricultural Sciences, Shandong, Jinan, China; 2 College of Plant Protection, Shandong Agricultural University, Shandong, Taian, China

**Keywords:** seed microbiome, core microbiome, 16S rRNA gene amplicon sequencing, *Sphingomonas endophytica*, microbial inoculant

## Abstract

The oilseeds are abundant in oils and proteins, and the production of high-quality oilseeds represents a major objective in modern agriculture. However, oilseed production is constrained by biotic and abiotic stresses, resulting in the decreasing in yield and quality. The seed microbiome has been recognized as a critical determinant of plant health. However, its composition and functional roles in various oilseed crops remains poorly explored. In this study, we utilized 16S rRNA gene amplicon sequencing to compare the bacterial component of seed microbiome and predict their metabolic potential in four oil crops (rapeseed, sunflower, sesame, soybean). Our results revealed that the oilseed harbored high diverse of microbes, and the assembly of microbial community was not random but driven by species and cultivar. From the perspective of microbial functions, the lipid metabolism and other secondary metabolites of seed microbes were associated with corresponding metabolic processes in seeds, such as glucosinolate and linoleic acid, reflecting the functional connection between seed metabolites and seed microbes. Furthermore, the core microbiome was obtained among four oilseed groups, consisting of 18 bacterial amplicon sequence variants (ASVs), including putative plant-beneficial resources, such as *Sphingomonas*. Notably, strain SE-S32 (*Sphingomonas endophytica*) isolated from rapeseed seed, one of the core microbes, could improve the resistance of various crops, indicating that seed core microbes could serve as a microbial inoculant among multiple crops. These results provide new insights into the correlation between seed microbiome and seed metabolites, establishing a theoretical foundation for developing microbial strategies to improve oilseed quality and plant health.

## 1 Introduction

Oilseed crops are indispensable components of agricultural production, serving as primary sources of edible oils, high-protein animal feed and industrial biofuels. Due to the increasing demand for the high content of oil and protein in oilseeds, the development of high-quality oilseeds has become strategically important (Zafar et al., 2019). However, emerging challenges including climate change, pathogen outbreaks, pest infestations and abiotic stresses pose serious threats to the yield and quality of oilseed production (Wheeler and von Braun, 2013; Savary et al., 2019; Ahmad et al., 2021; Zandalinas et al., 2021). There is an urgent need to develop innovative strategies to enhance the stress resistance of oilseed crops and improve oilseed quality.

The plant microbiome, which forms complex co-associations with plants (Trivedi et al., 2020), has attracted increasing attention due to its critical role in supporting plant nutrition (Zhang et al., 2019; Harbort et al., 2020; Yu et al., 2021), enhancing stress adaptation (Kwak et al., 2018; Carrión et al., 2019; Zhou et al., 2023) and modulating the traits of seed quality, including the content of protein, oil and glucosinolate (Han et al., 2024; Walsh et al., 2024; Hong et al., 2025). Plants provide a multitude of ecological niches for the colonization of microorganisms, encompassing the rhizosphere, root, leaf and seed. Specifically, research on plant microbiome mainly focuses on the rhizosphere, but seed microbiome is also worthy of attention (Matsumoto et al., 2021). Historically, seeds were once regarded as vectors for pathogenic microorganisms (Baker and Smith, 1966; Abdelfattah et al., 2023), however, they are also recognized as reservoirs of diverse microbial communities (Berg and Raaijmakers, 2018). The seed microbiome is receiving increasing attention from researchers and has recently emerged as a key factor of plant health (Romão et al., 2025). Representing an evolutionary legacy of plants, it constitutes a largely untapped resource for enhancing host fitness and resistance (Abdelfattah et al., 2023). Recent advances in characterizing the structure of seed microbiome have stimulated growing research interest in seed microbial communities (Bziuk et al., 2021; Simonin et al., 2022; Wassermann et al., 2022; Zhang et al., 2022b). Seed microbes contribute to plant fitness through multiple functional mechanisms, such as synthesizing hormones, solubilizing essential nutrients, and producing antimicrobial compounds (Shahzad et al., 2016; Yang et al., 2020; Shao et al., 2021; Romão et al., 2025). Their fundamental roles encompass facilitating seed germination (Liu et al., 2024; Li et al., 2025), regulating nutrient acquisition and promoting seedling growth (Mukherjee et al., 2020; Pal et al., 2024). Additionally, seed microbes,such as *Sphingomonas melonis*, *Bacillus velezensis* and *Bacillus altitudinis*, could improve plant resistance to disease and play a hidden role in the phytopathology paradigm of disease triangle (Chen et al., 2020b; Matsumoto et al., 2021; Wu and Chang, 2024). Furthermore, seed microbes such as *Pantoea deleyi* and *Bacillus* sp., mediate herbicide resistance evolution and enhance stress tolerance to antibiotics in seeds, which could reduce crops’ dependence on chemical herbicides and regulate antibiotic accumulation, respectively (Hu et al., 2024; Wang et al., 2025). Collectively, these studies highlight the importance of seed-associated microbes in plant fitness and their potential as sustainable agricultural resources.

Even though seeds are diverse and extremely variable, a portion of the microbial communities remains conserved within various seeds, referred to as the seed core microbiome (Simonin et al., 2022). It colonizes in seeds consistently and is thought to provide essential functions supporting seedling health and development (Romão et al., 2025). The persistence of core microbiome in seeds among species and cultivars suggests the co-evolutionary adaptation and functional indispensability (Simonin et al., 2022). Despite its potential ecological and practical importance, the core microbiome remains poorly characterized in variety oilseeds. Oilseeds are distinguished by high contents of lipids, proteins, and specialized metabolites, which likely impose strong selective pressures on their associated microbiome. Comparative analysis of the seed microbiome among multiple oilseed species offers a powerful opportunity to elucidate the relationship between microbial community structure and seed quality traits. Moreover, identifying the core microbiome among major oilseed crops would facilitate the selection of universal seed microbes, which are capable of enhancing key agronomic functions, such as nutrient acquisition, seedling growth, and stress tolerance (Qiu et al., 2019; Zhang et al., 2022a; Xia et al., 2024; Cao et al., 2025). In this study, seeds of four major oilseed crops (soybean, rapeseed, sesame, sunflower) were collected, all of them are cultivated worldwide. These four oilseed crops cover a wide range of genetic backgrounds and seed biochemical compositions, which provide seed microbes with distinctly different living environments. By using 16S rRNA gene amplicon sequencing, the structure of seed microbiome among multiple species and cultivars were characterized. We identified the core microbiome among the four oilseeds, consisting of a total of 18 ASVs, including microbial resources such as *Sphingomonas*. The functions of seed core microbes were also discovered. The strain SE-S32 (*Sphingomonas endophytica*), isolated from rapeseed seeds, could enhance the resistance of rapeseed and soybean seedlings to sclerotinia stem rot by seed inoculation. Furthermore, we predict the KEGG pathways associated with each seed microbiome using PICRUSt2, and identified the correlations between seed microbiome and seed metabolites. This study provides new insights into the relationship between microbial functions and seed metabolites, and offers an approach of utilizing seed core microbes to promote the health of various oilseed crops.

## 2 Materials and methods

### 2.1 The metadata of each cultivar among four oil crops

The seeds of sunflower, sesame and soybean were randomly purchased form commercial seed market with five cultivars of each oil crop. The metadata of each cultivar was provided in the **Supplementary Table S1**. After purchasing the oilseeds, we stored them at room temperature and complete DNA extraction for all samples within two weeks. To ensure the randomness of crop cultivars and avoid selection bias, cultivars are randomly selected without preference for specific biological phenotypes. Five cultivars were acquired of each species: sunflower (XC858, W8S-198, DW997, S9178, TY361), soybean (HuaiXianDou No.7, ZhongHuang No.13, ZhongHuang No.37, JiDou No.12, JiaoDa No.18) and sesame (HangZhi No.1, ZhuZhi No.28, YuZhi No.10, JuZhi No.1, HangTianHeiZhi No.16-8). The seed microbiome of rapeseed (HuaShuang No.4) was obtained from the previous study. The dynamic changes of seed microbiome crossing the entire seed life history were analyzed, including flower buds, young pods, developing seeds at 20, 30, 40, and 50 days after flowering, mature seeds and parental seeds (Yao et al., 2024). We assessed an analysis of the seed microbiome across multiple oilseed species and cultivars (soybean, sesame, sunflower), as well as the developmental succession within a single rapeseed seed cultivar.

### 2.2 DNA extraction, PCR amplification and 16S rRNA gene sequencing

To analyze the structure of seed endophytic microbiome, we performed surface sterilization on seeds to remove epiphytic microbes from seed surface. Overall, all of the oilseeds were surface sterilized (75% ethanol for 30s, 3.5% sodium hypochlorite for 1min, three times with sterilized water for 1 min each time). The sterilization control was verified by plating the final rinse water on R2A agar medium, and no microbial growth after 72 hours of inoculation confirmed the successful removal of surface contaminants, which indicated an epiphyte-free surface (Matsumoto et al., 2021). For each sequencing sample, ten oilseeds were randomly selected and pooled as one biological replicate. Furthermore, to ensure the reproducibility of the experiment, 16 biological replicates were taken from each oilseed cultivar. Then the oilseeds were mechanically disrupted using sterilized mortars and pestles. The consumables used during the DNA extraction and PCR process were sterilized by autoclaving (121°C, 30min) to minimize potential bacterial contamination from these sources. Total DNA was extracted from oilseeds by BioFast DNA for soil kit (Bioer Technology, China), followed by the manufacturer’s instructions. All DNA samples were stored in the 4 °C refrigerator and processed for subsequent experiments within one week. The V5–V7 regions of the 16S rRNA gene was performed by two-step PCR, which was according to the original research (Yao et al., 2024). DNA concentrations of oilseeds were measured by Nanodrop 1000 (Thermo Fisher Scientific, USA) and diluted to 20 ng/µL as PCR templates. The V5-V7 region was amplified with the primers 799F (Chelius and Triplett, 2001) and 1193R (Lundberg et al., 2012). The PCR program: 98 °C for 3 min; 30 cycles of 98 °C for 15 s, 55 °C for 15 s and 72 °C for 30 s, followed by 5 min at 72 °C. Each 50 µL PCR reaction contained 10 µL of 5 x buffer, 5 µL of dNTPs, 1 µL of DNA, 0.5 U of SuperNova DNA Polymerase (Bioer Technology, China), each forward and reverse primers at 10 nM, the remaining volume was supplemented with water. The amplification products were visualized via gel electrophoresis on 1.0% agarose. The total length of the PCR product, including the adapter sequences, is approximately 500 bp (Gao et al., 2024). We examined the negative control during PCR amplification based on the literature (Zhang et al., 2019). If no bands were visible in negative control (the PCR product without adding DNA template), the band at 500 bp target was purified by E.Z.N.A. Gel Extraction Kit (Omega Biotek, USA). The program of second-step PCR was the same as the first-step PCR, with only 8 cycles. If no amplification was visible in negative control, The PCR product of second-step PCR was purified by E.Z.N.A. Gel Extraction Kit (Omega Biotek, USA). Finally, the PCR products was mixed and the sequencing library was sent to the Company (Novogene, China). The NovaSeq 6000 platform was used to sequence the oilseed endophytic microbiome using paired end 250 bp strategy.

### 2.3 Bioinformatics analysis of 16S rRNA gene sequences

Oilseed endophytic microbiome was analyzed using QIIME2-2023.5 (Bolyen et al., 2019), followed by the official tutorials of QIIME2 (https://docs.qiime2.org/2023.5/tutorials/). The full commands of bioinformatics analysis, such as normalization steps and filtering thresholds, were provided in the **Additional File 2**, docx file. Paired-end reads were merged, denoised, and chimera-filtered using DADA2 to generate ASVs (Callahan et al., 2016). Taxonomic assignment of each ASV was performed by SILVA-138. The sequences classified as “chloroplast” and “mitochondria” were deleted through searching key words in QIIME2. Additionally, the sequences that were only annotated to the domain level of “bacteria” were also deleted. Finally, the feature table was normalized, and the subsequent analysis was performed using the normalized table. The final dataset comprised 77 samples for sunflower, 67 samples for soybean, 55 samples for rapeseed, and 80 samples for sesame, yielding a total of 279 sequencing samples. Alpha diversity was showed by the Shannon index at the ASV level. Beta-diversity was calculated based on Bray–Curtis distance. Permutational multivariate analysis of variance (PERMANOVA, Adonis) was used to evaluate whether there were differences in any two groups. The functions of seed microbiome were obtained using PICRUSt2 (Douglas et al., 2020) through an online website (https://www.bioincloud.tech/). Differential KEGG functions were analyzed by STAMP (Parks et al., 2014) and visualized in heatmap drawn by TB tools (Chen et al., 2020a). The core microbiome was defined as the ASVs that appear in more than 60% samples with in each crop. This threshold of 60% was selected as a balanced criterion to identify core ASVs that are both highly prevalent and specifically associated with oilseed species. The core microbiome was showed by Venn diagram (http://bioinformatics.psb.ugent.be/webtools/Venn/).

### 2.4 Plant treatments and assays of soybean seedlings in greenhouse

Seed endophyte SE-S32 (*Sphingomonas endophytica*) was isolated from rapeseed seed in the previous study (Yao et al., 2024). Inoculation soybean seeds with SE-S32 followed the method described by (Zhang et al., 2020). Briefly, soybean seeds were soaked in the bacterial suspension of SE-S32 for 4h, and the non-inoculated soybean seeds were treated with 0.01 M PBS solution. The details were provided below. The strain SE-S32 was cultured on R2A agar medium (Hopebio, China) for 3 days at 25 °C. The R2A agar medium was found to be free of contamination, containing only SE-S32 colony morphology. Subsequently, the inoculum concentration of SE-S32 was conducted. The R2A agar medium containing SE-S32 strain was washed with 0.01 M PBS solution to obtain the bacterial liquid, and the OD_600nm_ was diluted to 0.4 for microbial inoculation in soybean seeds. The surface-sterilized soybean seeds (cultivar: ZhongHuang No.13) were soaked in the SE-S32 bacterial liquid. 4 hours later, the soybean seeds were transferred to soil (Peilei Substrate Technology, China). Soybean seedlings grew in greenhouse with alternating light and dark for 14 h/10 h at 25 °C for 2 weeks.

### 2.5 Seedling growth and antagonistic activities against Sclerotinia stem rot

The assays of soybean seedling growth and antagonistic activities against Sclerotinia stem rot were performed by the method described by (Zhang et al., 2020). The fresh weight of aerial parts, dry weight of aerial parts and root length were calculated, respectively. Soybean seedlings were inoculated with *Sc*. *sclerotiorum* strain EP-1PNA367, a strain with highly virulent (Yu et al., 2010). It was cultured continuously in Potato Dextrose Agar (PDA) medium for 2 generations. Mycelial agar was inoculated onto leaves and the necrosis areas were counted after 36h to evaluate the resistance of soybean seedlings to *Sc*. *sclerotiorum*. Data analysis was based on one-way ANOVA (*p* value < 0.05) in Excel software. The seedling-related experiments were repeated three times with 6 seedlings per biological replicate. The experimental results showed consistent trends. The symbol “**” indicates *p* value < 0.01 and the symbol “NS” indicates *p* value > 0.05. The representative raw data was provided in **Supplementary Table S6**.

### 2.6 Antagonism assay between strain SE-S32 and EP-1PNA367

Strains SE-S32 and EP-1PNA367 were activated continuously for 2 generations on R2A and PDA agar plates (90 mm diameter), respectively, to ensure that the strains have strong vitality. Strain SE-S32 was activated at the edge of the 1/2 R2A agar plate to ensure early growth of the SE-S32 on the plate. Two days later, EP-1PNA367 (5 mm in diameter) was inoculated at the center of the culture dish. The culture dish inoculated with strain EP-1PNA367 alone served as the control. All treatments were incubated at 25 °C. After three days, the antagonistic activity was quantified by measuring the colony diameter of strain EP-1PNA367 in the direction toward the bacterial streak. Data analysis was based on one-way ANOVA (*p* value < 0.05) in Excel software. The symbol “*****” indicates *p* value < 0.00001. The representative raw data was provided in **Supplementary Table S6**.

## 3 Result

### 3.1 Alpha and beta diversity of seed microbiome among four major oilseed crops

**Figure 1:**
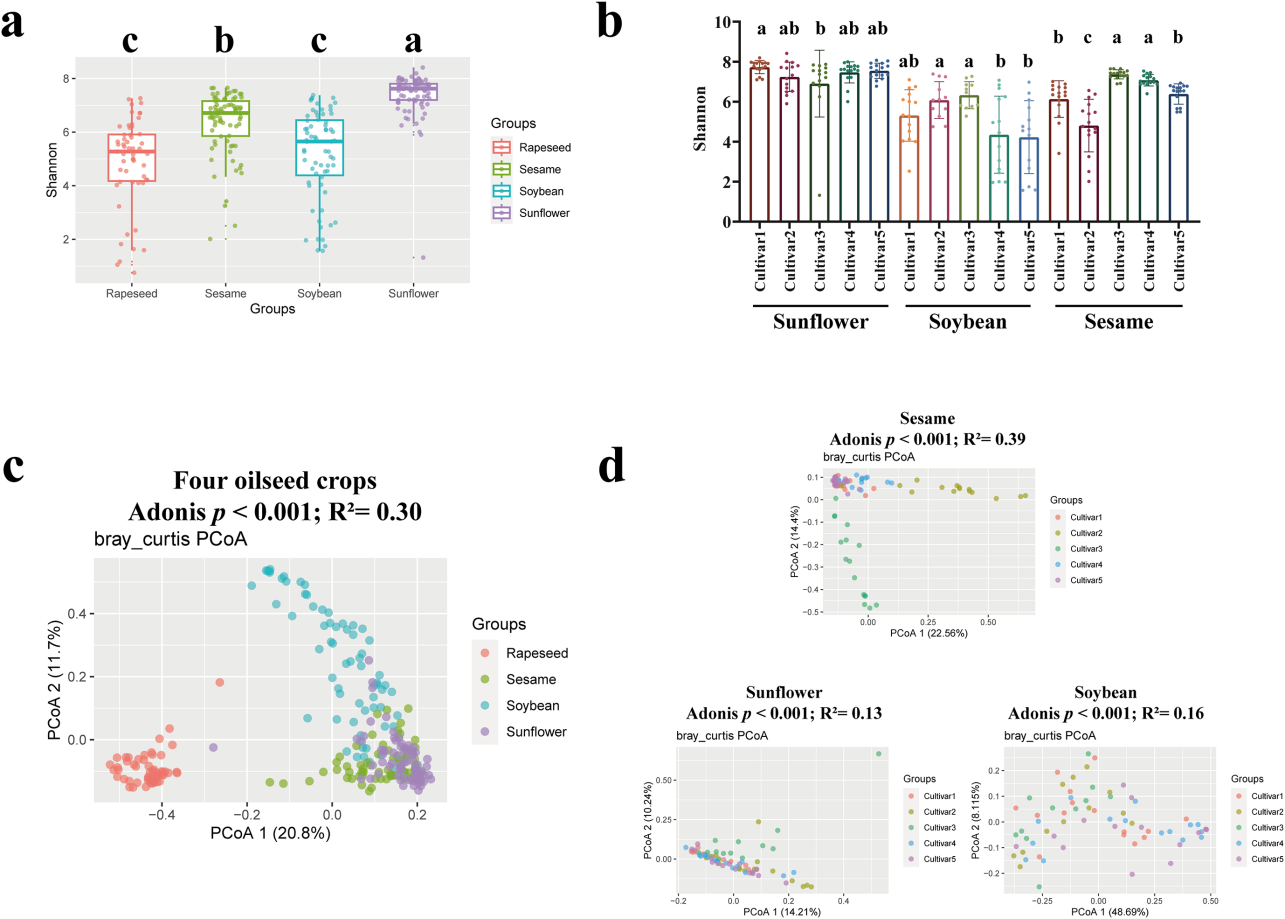
The microbial diversity of seed microbiome among four plant species. The Shannon index among all sequencing samples based on species **(a)** and cultivars **(b)**, respectively. Statistical significance was determined by one-way ANOVA (Duncan’s test). Letters (a–c) represent the significant differences at the 95% confidence interval. The horizontal line within boxes represents the value of medians, the tops of boxes represent the 75th percentiles and the bottoms of boxes represent the 25th percentiles. The upper and lower error bars extend to data no more than 1.5× interquartile range from the upper edge and lower edge of the box, respectively. **(c)** PCoA based on Bray–Curtis distance revealed the structure of seed microbiome among all oilseed samples. Statistical support from PERMANOVA indicated that different species explained a significant portion of the variance (*p* value < 0.001, reported as the raw *p* value without multiple-test correction; R²=0.30). **(d)** PCoA were sequentially conducted for different cultivars within species for sesame, sunflower, and soybean. Permutational multivariate analysis of variance (PERMANOVA, Adonis) was used to test whether there were differences between different groups. Sesame (R² = 0.39, *p* value < 0.001), sunflower (R² = 0.13, *p* value < 0.001), and soybean (R² = 0.16, *p* value < 0.001).

To assess the structure of seed microbiome among major oilseed crops, we conducted 16S rRNA gene sequencing on seeds from four species (soybean, sunflower, sesame, rapeseed). This approach provided a cross-species and multi-cultivar overview of the oilseed endophytic microbiome. Seeds of soybean, sesame, and sunflower were purchased from the seed markets, with five cultivars selected for each species. The structure of rapeseed seeds microbiome was sourced from the previous study (Yao et al., 2024), encompassing the dynamic changes during seed development. Overall, a total of 16284 bacterial amplicon sequence variants (ASVs) were identified among 279 oilseed sequencing samples, indicating that oilseeds harbor rich and diverse microbial resources. To evaluate the microbial diversity of each seed microbiome, the Shannon index was calculated at the ASV level (**Figure 1a**; **Supplementary Table S2**). Sunflower seeds exhibited the highest Shannon index, followed by rapeseed and sesame, whereas soybean showed the lowest number (one-way ANOVA, *p* < 0.05). Moreover, significant differences in Shannon index were observed among different cultivars (**Figure 1b**). Principal coordinates analysis (PCoA) was carried out to reveal the differences in the structure of seed microbiome, and the statistical method of permutational multivariate analysis of variance (PERMANOVA, Adonis) was used to test whether there were differences between different species (**Figure 1c**) or cultivars (**Figure 1d**). The *p* value between different species or cultivars was less than 0.05, and the R² value was more than 0.13, suggesting that the assembly of seed microbiome was not random, each species and cultivar could form its own unique microbial community. Furthermore, there was an overlap in the sequencing samples analyzed by PCoA, indicating the existence of a conserved microbiome among different oilseed species.

### 3.2 Taxonomic composition of seed microbiome at the phylum and genus levels

**Figure 2:**
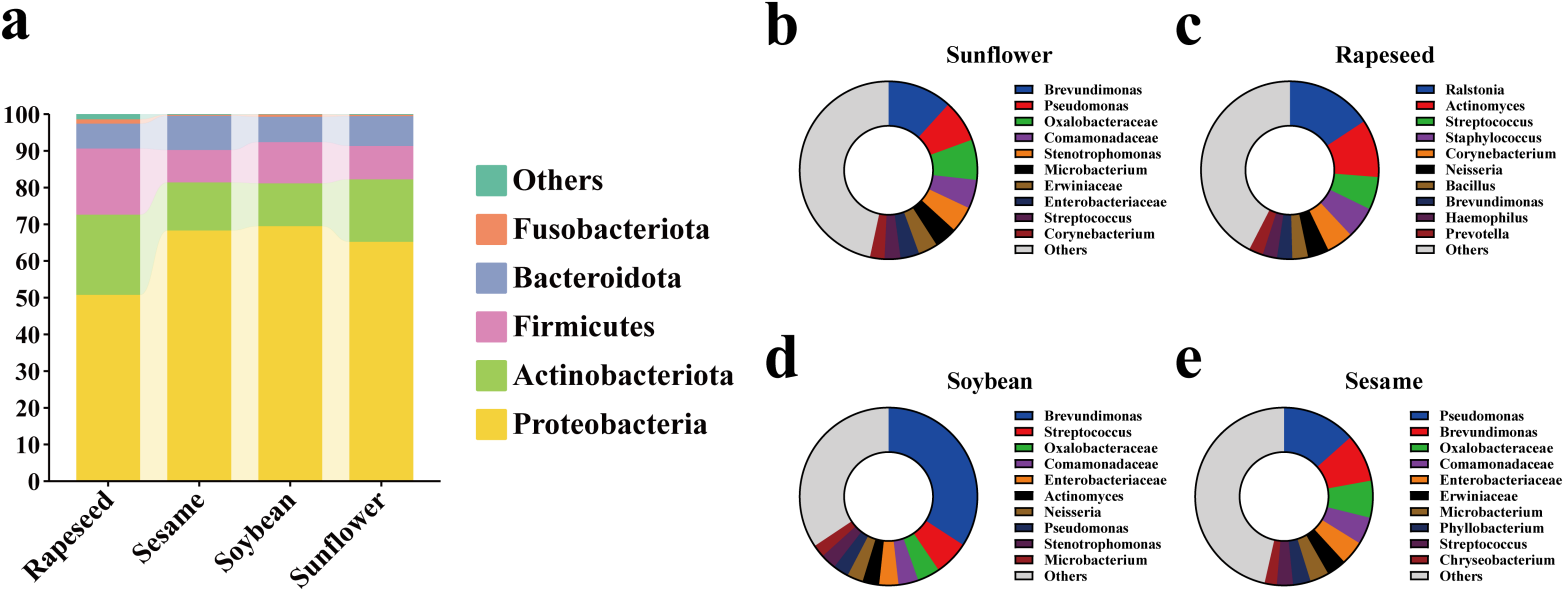
The composition of seed microbiome of four oil crops at the phylum and genus levels, respectively. **(a)** The relative abundances in sunflower, soybean, rapeseed and sesame seeds at the phylum level. The top 10 genera in relative abundance are shown in the diagram among sunflower **(b)**, soybean **(c)**, rapeseed **(d)**, and sesame **(e)**, respectively.

To investigate the composition of the seed microbiome among the four oilseed crops, we analyzed the microbial communities at the phylum and genus levels, respectively. The seed microbiome of all oilseeds was predominantly dominated by Proteobacteria, with relative abundances ranging from 50.78% to 69.49%, followed by Actinobacteriota, Firmicutes, Bacteroidota, and Fusobacteriota. These five phyla accounted for over 98.63% of the total relative abundance, representing the absolute dominant taxonomic groups within the seed microbiome (**Figure 2a**). Furthermore, the top 10 genera in relative abundance within each oilseed crop were identified, and each oilseed crop exhibited a distinct genus-level microbial composition. The ASVs that could not be annotated at the genus level were described using the taxonomy of the previous taxonomic level. The genera with the highest abundance in sunflower and sesame seeds were *Brevundimonas*, *Pseudomonas* and Oxalobacteraceae. The dominant genera were *Ralstonia*, *Actinomyces* and *Streptococcus* in rapeseed seeds. While soybean seeds showed high abundances of *Brevundimonas*, *Streptococcus* and Oxalobacteraceae (**Figures 2b–e**; **Supplementary Table S3**).

### 3.3 Identification of the core and unique microbiome of four major oilseed crops

**Figure 3:**
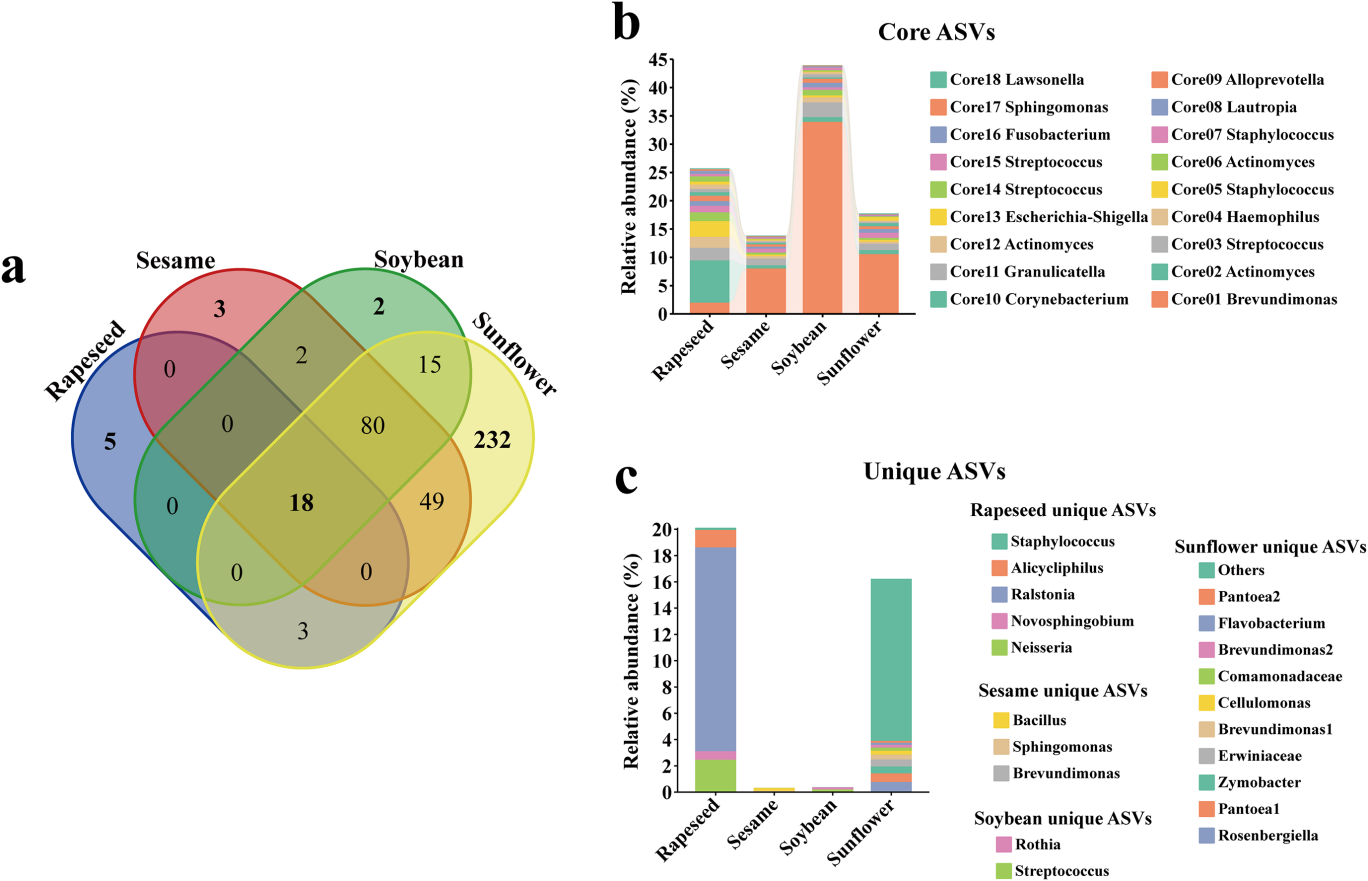
Identification of core seed microbiome of four oilseed crops. **(a)** The Venn diagram showing that a total of 18 core ASVs coexist within all groups. **(b)** The composition and relative abundance of 18 core ASVs among all groups. **(c)** The relative abundance of unique ASVs among all oilseed crops. Rapeseed, Sesame, Soybean, and Sunflower represented the core microbiome of this species. The unique ASVs represented the core ASVs present in specific species.

Despite variations in microbial communities among different crops and cultivars, a core microbiome was consistently identified in all host groups. In this study, the core microbiome was defined as the ASVs detected in at least 60% sequencing samples within each crop. We also analyzed how the results changed under other thresholds. As anticipated, a higher threshold (75%) might be too stringent, identifying only 11 core ASVs. Meaningful core members may be excluded, which are consistently present but affected by technical factors or numbers of sequencing samples. Conversely, a lower threshold (50%) yielded 29 ASVs, which may include common but less host-specific ASVs. Among all 16284 ASVs, there were 18 ASVs existing in all four oilseed groups, indicating that a subset of the microbes could coexist stably in multiple species (**Figure 3a**; **Supplementary Table S4**). Overall, the total relative abundance of the core microbiome accounted for 13.84-43.95%. These conserved ASVs encompassed microbial resources with presumed plant-beneficial functions, such as *Sphingomonas* (Matsumoto et al., 2021; Li et al., 2022; Yin et al., 2022; Xu et al., 2023) and *Brevundimonas* (Brachi et al., 2022), as well as microbes whose functional roles in plants remain poorly characterized, including *Fusobacterium* and *Haemophilus* (**Figure 3b**). Some microbes that may be associated with human-related environments or clinical contexts, including *Lawsonella* (Odendaal et al., 2024) and *Streptococcus* (Tap et al., 2023). Additionally, several ASVs exhibited host specificity. For instance, ASV *Ralstonia* was predominantly associated with rapeseed, ASV *Bacillus* with sesame and ASV *Rothia* with soybean (**Figure 3c**; **Supplementary Table S4**). These ASVs were present within the core microbiome of respective host, but they were absent or rare in others, indicating that their assembly is strongly influenced by host-specific factors, which ultimately restrict their occurrence to particular species.

### 3.4 The predictive functions of seed microbiome of four major oilseed crops

**Figure 4:**
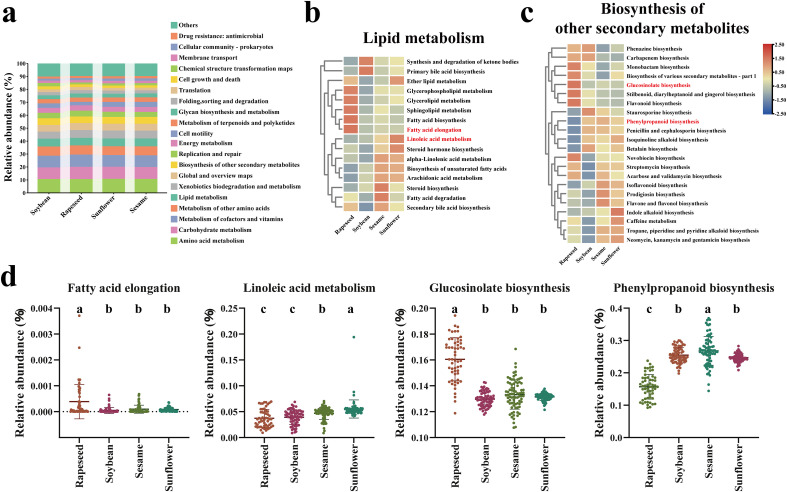
The functions of seed microbiome of four oilseed crops based on PICRUSt2. **(a)** The prediction of the top 20 KEGG pathways in each group at the second level. Heatmap showing the differential functions among all groups of KEGG pathways at the third level within lipid metabolism **(b)** and biosynthesis of other secondary metabolites **(c)**, respectively. **(d)** Partial differential KEGG pathways among four oilseed crops (fatty acid elongation, linoleic acid metabolism, glucosinolate biosynthesis and phenylpropanoid biosynthesis). The differential pathways were analyzed through STAMP, one-way ANOVA followed by Tukey-Kramer test was applied for multi-group comparisons (*p* value < 0.05).

To investigate the correlation between the seed microbiome and host metabolic functions, we performed functional prediction of the microbial communities using PICRUSt2. At the second level of KEGG analysis, the predicted functions of the seed microbiome among all four oilseed crops were primarily dominated by amino acid metabolism, carbohydrate metabolism and metabolism of cofactors and vitamins (**Figure 4a**; **Supplementary Table S5**). Given the central role of lipid metabolism in oilseeds and the species-specific synthesis of metabolites, we further analyzed differential KEGG pathways at third level within the lipid metabolism (**Figure 4b**) and biosynthesis of other secondary metabolites (**Figure 4c**). The differential pathways were analyzed through STAMP (one way ANOVA, *p* value < 0.05) (Parks et al., 2014). The functions of rapeseed seed microbiome were inferred to exhibit significantly higher relative abundance in fatty acid elongation and glucosinolate biosynthesis than the other three groups (**Figures 4b, c**; **Supplementary Table S5**). Soybean seeds, characterized by relatively low oil content, showed the lowest relative abundance in multiple lipid metabolism pathways, including glycerolipid metabolism and fatty acid biosynthesis (**Figure 4b**). Sunflower seeds, recognized for the high content of linoleic acid (Li et al., 2024b), were predicted to be enriched in microbial functions related to linoleic acid metabolism. Additionally, sesame seeds could synthesize unique metabolites, such as sesamin and sesamolin (Li et al., 2024a), which may explain the predicted enrichment in phenylpropanoid biosynthesis (**Figure 4d**).

### 3.5 The core microbes enhance the resistance of various oilseed crops

**Figure 5:**
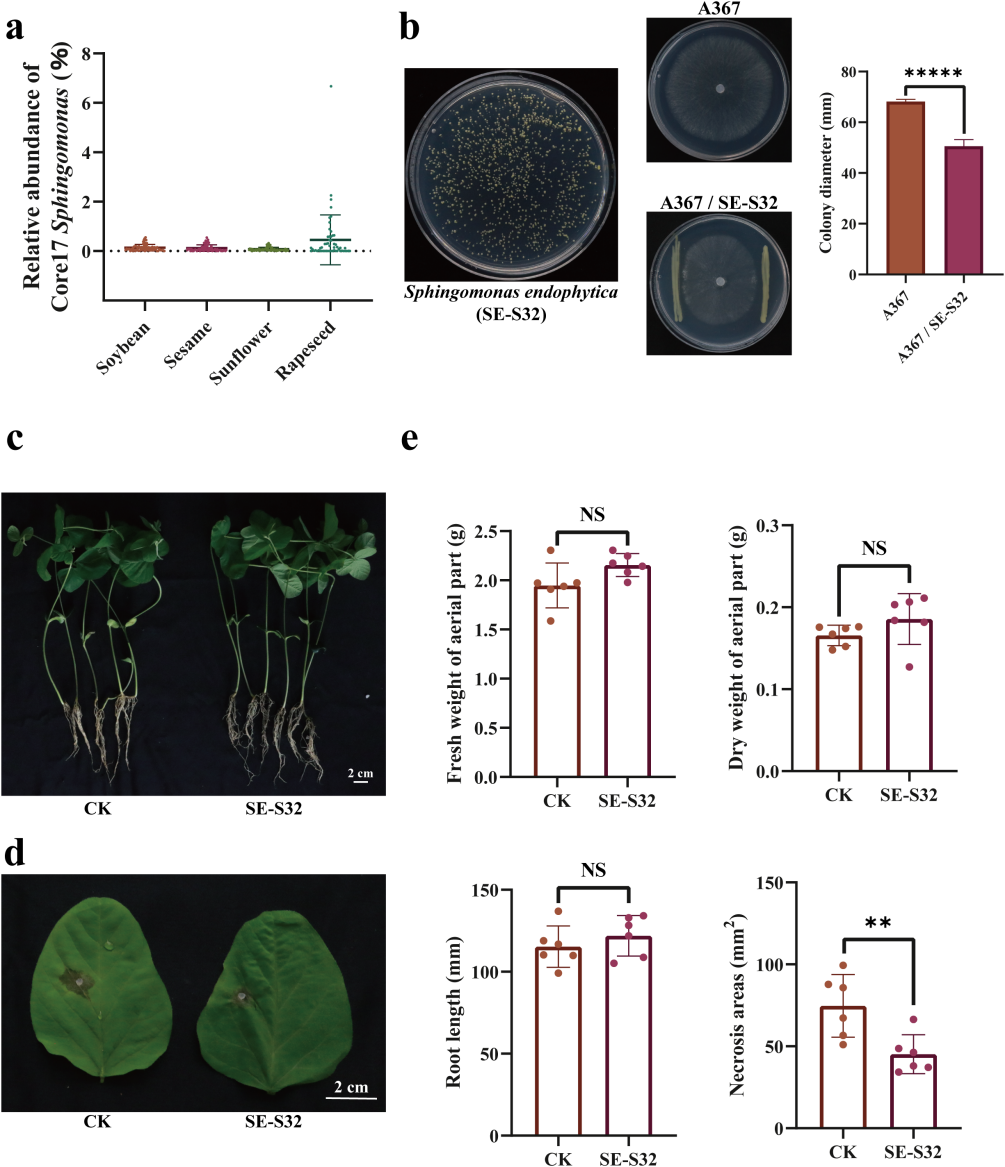
The morphology of strain SE-S32 and the effect of SE-S32 inoculation on soybean seedlings. **(a)** The relative abundance of ASV Core17 *Sphingomonas* in seed microbiome among four oilseed crops. **(b)** The morphology of strain SE-S32 was developed on R2A medium for 5 days under 25 °C, and its antagonistic effect on strain EP-1PNA367 (*Sc. sclerotiorum*). **(c)** The morphology of soybean seedlings growing in soil for two weeks under 25 °C, to compare the differences between the SE-S32 inoculated group and the control group. **(d)** The lesion size of soybean leaves after inoculation with 2-mm diameter hyphal disc of strain EP-1PNA367 (*Sc*. *Sclerotiorum*) for 36 hours, in the greenhouse under 25 °C. **(e)** Statistical data of fresh weight of aerial parts, dry weight of aerial parts, root length and lesion size of *Sc*. *Sclerotiorum* in leaves, respectively. Seedlings belonging to SE-S32 inoculation group are on the right. The seedlings belonging to the control group are on the left. The data are shown as the mean ± S.D. CK represents PBS inoculation; SE-S32 represents SE-S32 inoculation; A367 represents the control group inoculated with strain EP-1PNA367 alone; A367/SE-S32 represents the treatment group for the antagonism assay between SE-S32 and EP-1PNA367. Data analysis was based on one-way ANOVA. The symbol “**” indicates *p* < 0.01, the symbol “*****” indicates *p* < 0.00001, and the symbol “NS” indicates “no significance”.

Following identification of the core microbiome among four oilseed crops, we focused our research on seed core microbes to assess their potential functions in multiple plants. The ASV Core17 *Sphingomonas* was selected for investigation due to its high prevalence and conservative relative abundance. This ASV was one of the core members in the seed microbiome, with the relative abundance of 0.08%-0.46% among the four oilseed crops (**Figure 5a**). It was detected in 238 out of 279 sequencing samples, corresponding to an occurrence frequency of 85%. We isolated an endophytic strain SE-S32 (*Sphingomonas endophytica*) from surface-sterilized rapeseed seeds (**Figure 5b**), the similarity of 16S rRNA gene sequence between SE-S32 and ASV Core17 *Sphingomonas* was 98.41% (372/378bp), and the inoculation with SE-S32 significantly promoted the growth of rapeseed seedlings and enhanced its resistance to Sclerotinia stem rot (Yao et al., 2024), a major disease of rapeseed caused by *Sclerotinia sclerotiorum*. Furthermore, antagonistic assays on R2A medium revealed that SE-S32 significantly suppressed the growth of strain EP-1PNA367 (*Sc. sclerotiorum*), with a reduction in colony diameter of 26% (**Figure 5b**). To assess its potential as a cross‐species beneficial microbe, SE-S32 was inoculated into soybean seeds, and its effects on biological phenotypes were evaluated in 14-day seedlings. The experimental results showed that inoculation with SE-S32 had no adverse effect on soybean growth, no significant differences were observed in the fresh weight of aerial weight, the dry weight of aerial weight and root length between the SE-S32 inoculated group and the control group (**Figures 5c, e**). We further explored the resistance of soybean seedlings to Sclerotinia stem rot. It was found that the SE-S32 inoculated group had significantly lower lesion area than the control group, with a 39% reduction in lesion area (**Figures 5d, e**).

## 4 Discussion

This study provides a comprehensive analysis of the seed microbiome among four major oilseed crops (soybean, rapeseed, sunflower, and sesame), focusing on the bacterial component, revealing that the assembly of seed-associated microbial communities is not random but shaped by both host species and genotype. The specialized metabolites of each oilseed, such as glucosinolates in rapeseed, appear to select for specific seed microbial communities (Unger et al., 2024). Our findings align with the recognition that host genotype and seed biochemical characteristics serve as the filtering mechanisms in microbial assembly (Zhang et al., 2019; Brachi et al., 2022). This host-mediated selection mechanism likely drives functional adaptations in seed microbial communities, potentially enhancing compatibility between seed microbes and seed development.

**The predicted metabolites of oilseed microbes are involved in corresponding metabolites in oilseeds.** The significance of metabolites has gained increasing attention in oilseeds research (Kefale et al., 2024). From the perspective of microbial function, it was predicted by PICRUSt2 and displayed by KEGG pathway (Douglas et al., 2020). Microbial functions associated with glycerolipid metabolism and fatty acid biosynthesis were detected among all four crops, indicating that microbes may participate in oil accumulation, which is one of the most important traits in oilseeds. Furthermore, the predictions revealed that the lipids and secondary metabolites of seed microbiome are consistent with the corresponding metabolites in seeds. This is evident in rapeseed seeds, which could produce unique metabolites compared to the other three species, such as glucosinolates and erucic acid (Harvey and Downey, 1964; Seo and Kim, 2017; Xu et al., 2024). Glucosinolates serve as the primary defense compounds in Brassicaceae (Seo and Kim, 2017), while erucic acid is a product of fatty acid elongation (Lassner et al., 1996). Sunflower seeds, as a rich source of linoleic acid, harbored the seed microbiome with higher level of linoleic acid metabolism (Li et al., 2024b). These results indicated a correlation between the microbial functions and the functions within hosts, and seed microbes may participate in biochemical processes, the conclusion is consistent with the above research (Yao et al., 2024). Although these findings are predictive, they provide evidence for the positive role of microbes in seed quality and adaptability. Validation through isolated bacteria is needed to confirm the interaction and relationship between these functional strains and seed quality. Furthermore, the excellent characteristics of a cultivar may be contributed by its related microbes, providing a new research perspective for obtaining seeds with high quality.

**A conserved core microbiome exists in oilseed crops.** Despite significant variations in the composition of microbial community among species and cultivars, the identification of the core microbiome across the four major oilseed crops (soybean, rapeseed, sunflower, sesame) represents a key finding in this study. By analyzing seed microbiome across multiple crop cultivars (sunflower, soybean, sesame) and developmental stages (rapeseed), a more stringent structure of core microbiome with functional potential was obtained, which may play a crucial role in plant health. The consistent presence of 18 core ASVs among all hosts suggests that these microbes form a stable, conserved holobiont with oilseeds. This persistence of these microbes indicates the possibility of co-evolution and functional indispensability between hosts and microbes, and core microbes may provide essential functions critical for seed quality. Notably, the core ASVs such as *Sphingomonas* and *Brevundimonas*, have been previously implicated in plant growth promotion and stress tolerance, suggesting their potential roles in maintaining seedling health among multiple species (Matsumoto et al., 2021; Brachi et al., 2022; Li et al., 2022; Yin et al., 2022; Xu et al., 2023). Conversely, other conserved ASVs including *Fusobacterium* and *Haemophilus*, whose ecological roles in plants remain poorly understood, were detected among oilseeds. These microbes highlight knowledge gaps in seed microbiome, and emphasizes the need for more functional features in addition to commonly phenotypes, such as promoting plant growth. Furthermore, the core ASV *Streptococcus* is associated with human-related environments (Tap et al., 2023; Odendaal et al., 2024) and defined as microbes found across all habitats, including human gut, plant and soil (Ma et al., 2025). The importance of plant microbiome in human health is increasingly recognized and identifying potential cross-kingdom microbiome is essential for achieving better “One Health” outcomes (Singh et al., 2023). The presence of seed core microbiome suggests that they may adapt to survive in seed, human gut or environment, and play multiple roles in various habitats.

Seed core microbes exhibit beneficial functions among various crops. In particular, the functional study of seed core microbe SE-S32 (*Sphingomonas endophytica*) were calculated, which enhanced disease resistance in both rapeseed and soybean seedlings toward Sclerotinia stem rot, one of the major diseases affecting rapeseed and soybean production (Talmo and Ranjan, 2025). These results highlight the functional adaptability of seed core microbes in different crops. Although SE-S32 is a core member in seed microbiome, its natural relative abundance in seeds is relatively low. A reference related to endophyte in rice seeds indicated that *Sphingomonas* was present in both disease-resistant and susceptible phenotypes, but its abundance was significantly higher in disease-resistant rice seeds (Matsumoto et al., 2021). It is speculated that exogenous inoculation ensures the establishment of SE-S32 during the early stages of seedling development, thereby effectively enhancing the naturally occurring but inefficient disease-resistance of oilseed crops. This cross-host efficacy highlights the potential of seed core microbes as universal biological inoculants applicable to multiple oilseed crops, supporting sustainable agricultural development. Regarding the mechanism by which SE-S32 inhibits EP-1PNA367 growth, the strain SE-32 could directly inhibit the growth of EP-1PNA367. This effect is hypothesized to result from nutrient competition and the production of antibiotic compounds. Beyond this direct antagonism, the impact of SE-S32 inoculation on plants is equally worthy of studying. To confirm that the cross-species resistance is driven by SE-S32 colonization, future studies employing fluorescent tagging will be necessary to directly visualize the colonization of SE-S32 in multiple plants. In addition, the effects of SE-S32 on host defense-related pathways, such as the induction of plant defense responses, are equally crucial and warrants investigation. Furthermore, some unique ASVs exhibited host specificity, such as *Ralstonia* in rapeseed seeds, *Bacillus* in sesame seeds and *Pantoea* in sunflower seeds. Studies have demonstrated their beneficial functions for crops (Wei et al., 2013; Seo and Kim, 2017; Berlanga-Clavero et al., 2022; Xu et al., 2022), There may be specific effects of these unique microbes in certain oilseed crops, and further research is needed.

Overall, the structure of seed microbiome was obtained among four major oilseed crops and their main cultivars. We demonstrated the correlation between microbial function and host metabolism, and proposed a microbial-mediated approach to improve oilseed quality and seedling resistance. Consider that core microbes could coexist compatibly within variety oilseeds and interact synergistically with plants, future research should focus on functional validation of 18 core ASVs and elucidate the common mechanisms across species. It is imperative to investigate the mechanisms by which core microbes influence plants. This research will pave the way for developing novel seed bioinoculants to enhance the seed quality and seedling health among variety crops.

## Data Availability

The datasets presented in this study can be found in online repositories. The names of the repository/repositories and accession number(s) can be found in the article/**Supplementary Material**.

## Supplementary material

Supplementary Table 1 The metadata of each sequencing samples of each cultivar among four oilseed crops.

Supplementary Table 2 The Shannon index of each group.

Supplementary Table 3 The compositions of seed microbiome at the phylum and genus levels.

Supplementary Table 4 The composition of core and unique ASVs.

Supplementary Table 5 The predictive microbial functions among four oilseed crops.

Supplementary Table 6 The raw data from biological experiments used in **Figure 5**. The **Additional File 2** contains the code used in QIIME2.
