# Supplementary material for: Comparative analysis of the seed microbiome in four major oilseed crops (rapeseed, sunflower, soybean, sesame) reveals host-specific assembly and potential application of seed core microbes
Source: Front Plant Sci. 2026 Jan 26;17:1721916. doi: 10.3389/fpls.2026.1721916 (PMC12883838; doi:10.3389/fpls.2026.1721916)
Supplement: Supplementary file 2 [file Table2.doc]

**1 Input the raw data of all sequencing samples**

qiime tools import \

--type 'SampleData[PairedEndSequencesWithQuality]' \

--input-path manifest \

--output-path paired-end-demux.qza \

--input-format PairedEndFastqManifestPhred33

**2** **Perform forward and reverse sequences assembly using datda2 and primer sequences**

qiime dada2 denoise-paired \

--i-demultiplexed-seqs paired-end-demux.qza \

--p-n-threads 50 \

--p-trim-left-f 19 \

--p-trim-left-r 18 \

--p-trunc-len-f 250 \

--p-trunc-len-r 250 \

--o-table table.qza \

--o-representative-sequences rep-seqs.qza \

--o-denoising-stats denoising-stats.qza

**3 Perform annotation on the assembled ASVs**

qiime feature-classifier classify-sklearn \

--i-classifier silva-138-99-nb-classifier.qza \

--i-reads rep-seqs.qza \

--o-classification taxonomy.qza

**4 Exclude chloroplast and mitochondria sequences**

qiime taxa filter-table \

--i-table table.qza \

--i-taxonomy taxonomy.qza \

--p-exclude chloroplast \

--o-filtered-table a-table.qza

qiime taxa filter-table \

--i-table a-table.qza \

--i-taxonomy taxonomy.qza \

--p-exclude mitochondria \

--o-filtered-table b-table.qza

**5** **Retain at least ASVs annotated to the phylum level**

qiime taxa filter-table \

--i-tableb-table.qza \

--i-taxonomy taxonomy.qza \

--p-include p__ \

--o-filtered-table c-table.qza

**6 Retain ASVs with a total frequency greater than 5**

qiime feature-table filter-features \

--i-table c-table.qza \

--p-min-frequency 5 \

--o-filtered-table d-table.qza

**7 Retain ASVs present in at least two sequencing samples**

qiime feature-table filter-features \

--i-table d-table.qza \

--p-min-samples 2 \

--o-filtered-table e-table.qza

**8 All samples were rarefied to the minimum sequence count to obtain a normalized table**

qiime feature-table rarefy \

--i-table e-table.qza \

--p-sampling-depth **minimum sequence** \

--o-rarefied-table normalized-table.qza

**9 Remove sequences without corresponding id in the normalized-table**

qiime feature-table filter-seqs \

--i-data rep-seqs.qza \

--i-table normalized-table.qza \

--p-no-exclude-ids \

--o-filtered-data normalized-rep-seqs.qza

**10 Generate ASV tables at the phylum and genus levels**

qiime taxa collapse \

--i-table normalized-table.qza \

--i-taxonomy taxonomy.qza\

--p-level 2\

--o-collapsed-table lv2-table.qza

qiime taxa collapse \

--i-table normalized-table.qza \

--i-taxonomy taxonomy.qza\

--p-level 6\

--o-collapsed-table lv6-table.qza

**11 Generate the Shannon index among each sample**

qiime diversity alpha \

--i-table normalized-table.qza \

--p-metric shannon \

--o-alpha-diversity shannon.qza

**12 Output various data in this study**

qiime tools export \

--input-path shannon.qza \

--output-path shannon

qiime tools export \

--input-path normalized-table.qza \

--output-path normalized-table

qiime tools export \

--input-path lv2-table.qza \

--output-path lv2-table

qiime tools export \

--input-path lv6-table.qza \

--output-path lv6-table

qiime tools export \

--input-path normalized-rep-seqs.qza \

--output-path normalized-rep-seqs
